# Potential anti‐SARS‐CoV‐2 drug candidates identified through virtual screening of the ChEMBL database for compounds that target the main coronavirus protease

**DOI:** 10.1002/2211-5463.12875

**Published:** 2020-05-29

**Authors:** Motonori Tsuji

**Affiliations:** ^1^ Institute of Molecular Function Misato‐shi, Saitama Japan

**Keywords:** 2019 novel coronavirus, COVID‐19, drug repositioning, M^pro^, SARS‐CoV‐2, virtual screening

## Abstract

A novel coronavirus [severe acute respiratory syndrome coronavirus 2 (SARS‐CoV‐2), or 2019 novel coronavirus] has been identified as the pathogen of coronavirus disease 2019. The main protease (M^pro^, also called 3‐chymotrypsin‐like protease) of SARS‐CoV‐2 is a potential target for treatment of COVID‐19. A M^pro^ homodimer structure suitable for docking simulations was prepared using a crystal structure (PDB ID: https://doi.org/10.2210/pdb6Y2G/pdb; resolution 2.20 Å). Structural refinement was performed in the presence of peptidomimetic α‐ketoamide inhibitors, which were previously disconnected from each Cys145 of the M^pro^ homodimer, and energy calculations were performed. Structure‐based virtual screenings were performed using the ChEMBL database. Through a total of 1 485 144 screenings, 64 potential drugs (11 approved, 14 clinical, and 39 preclinical drugs) were predicted to show high binding affinity with M^pro^. Additional docking simulations for predicted compounds with high binding affinity with M^pro^ suggested that 28 bioactive compounds may have potential as effective anti‐SARS‐CoV‐2 drug candidates. The procedure used in this study is a possible strategy for discovering anti‐SARS‐CoV‐2 drugs from drug libraries that may significantly shorten the clinical development period with regard to drug repositioning.

## Graphical abstract

Here, I describe virtual screenings for compounds targeting main protease of severe acute respiratory syndrome coronavirus 2 (SARS‐CoV‐2) to predict potential anti‐SARS‐CoV‐2 drugs and lead compounds. The procedure described herein may be a suitable drug repositioning strategy for the discovery of anti‐SARS‐CoV‐2 drugs from drug libraries.

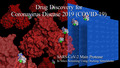

**Abbreviations**
M^pro^: main protease
SARS‐CoV‐2: severe acute respiratory syndrome coronavirus 2

A novel coronavirus (severe acute respiratory syndrome coronavirus 2 [SARS‐CoV‐2], or 2019 novel coronavirus [2019‐nCoV]) has been identified as the pathogen of coronavirus disease 2019. The coronavirus has spread worldwide and exhibits strong contagious and infective characteristics. There are no effective anti‐SARS‐CoV‐2 drugs at the time of writing. The discovery of potential anti‐SARS‐CoV‐2 drugs from known drug libraries is thought to be an effective drug repositioning strategy for shortening the clinical development period.

Severe acute respiratory syndrome coronavirus 2 belongs to the betacoronavirus group. One of the best‐characterized drug targets among viral constituents is the main protease (M^pro^, also called 3‐chymotrypsin‐like protease) [1]. Crystal structures of the M^pro^ dimer, the biological active form, have been resolved with or without synthetic inhibitors, some of which covalently bind to Cys145 at the catalytic dyad (i.e., Cys145 and His41) or have been designed to covalently bind to Cys145 (Fig. 1) [2, 3]. However, side effects, toxicity, and lower potency often cause covalent inhibitors to drop out. Therefore, noncovalent inhibitors with high binding affinity are more suited for treatment of such viral infections.

**Fig. 1:**
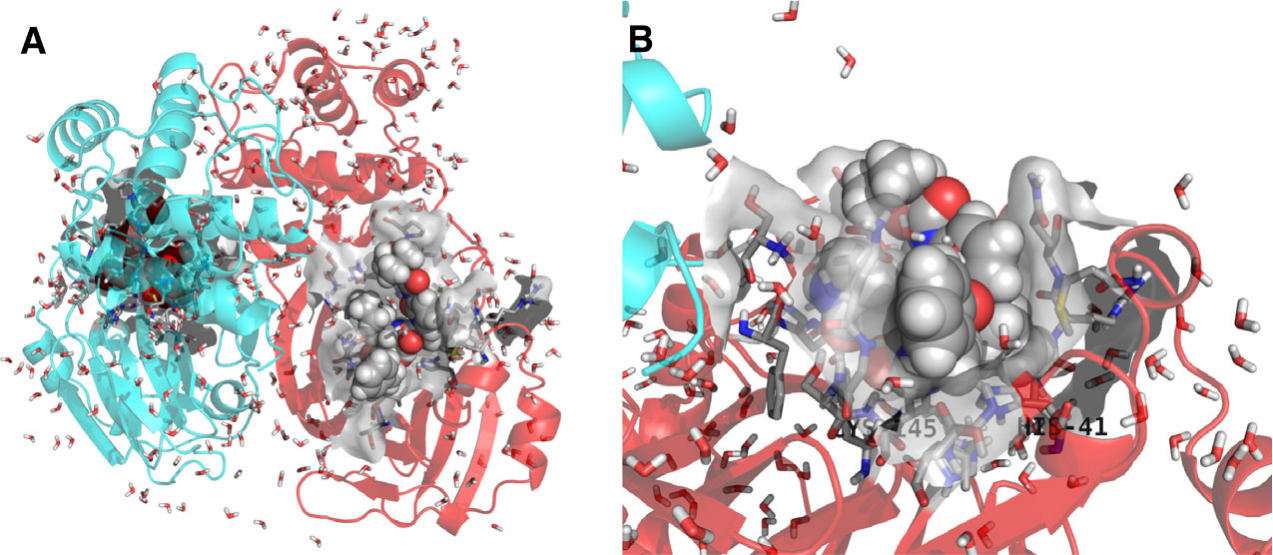
Refined crystal structure (PDB ID: https://doi.org/10.2210/pdb6Y2G/pdb) of a SARS‐CoV‐2 M^pro^ homodimer with peptidomimetic α‐ketoamide inhibitors. (A) Whole structure and (B) enlarged structure of the active site. Chains A and B of the M^pro^ homodimer are shown as red and cyan ribbons, respectively. Peptidomimetic α‐ketoamide inhibitors are shown in CPK color using space‐filling models. Water molecules are shown using tubes. Residues located 3 Å from the inhibitors are shown in CPK color using tubes without nonpolar hydrogen atoms. Van der Waals surfaces of the active sites are shown in gray.

In this study, I performed stepwise structure‐based virtual screenings using two different docking simulations in order to discover potential drugs that target M^pro^ using the ChEMBL database [4], which mainly lists drugs and known bioactive compounds. I present these potential anti‐SARS‐CoV‐2 drug candidates here. The structural information of the potential drugs will be useful for improving their pharmacokinetic properties more effectively and for developing specific anti‐SARS‐CoV‐2 drugs.

## Methods

### Refinement of the M^pro^ crystal structure

I prepared the M^pro^ homodimer structure suitable for docking simulations using a crystal structure (PDB ID: https://doi.org/10.2210/pdb6Y2G/pdb [2]; resolution 2.20 Å). Structural refinement was performed using Homology Modeling Professional for hyperchem (HMHC) software [5, 6], and energy calculations were performed under the AMBER99 force field with the following parameters: root‐mean‐square gradient, 1.0 kcal·mol^−1^·Å^−1^; algorithm, Polak–Ribière; cutoffs, none; 1–4 van der Waals scale factor, 0.5; 1–4 electrostatic scale factor, 0.833; dielectric scale factor, 1.0; and distance‐dependent dielectric condition. Structural refinement was conducted in the presence of peptidomimetic α‐ketoamide inhibitors, which were previously disconnected from each Cys145 of the M^pro^ homodimer. After adding hydrogen atoms automatically, I assigned Mulliken atomic charges of inhibitors; their α‐carbonyl moiety (disconnected from Cys145) was treated as a hydroxy carbocation using single‐point calculations of the semiempirical MNDO/d method. Mulliken atomic charges obtained from the MNDO/d calculation showed empirically good correlation with AMBER charges [7]. In addition, AMBER99 atom types were assigned. N‐ and C‐terminals of the M^pro^ homodimer were treated as zwitterions, aspartic and glutamic acid residues were treated as anions, while lysine, arginine, and histidine residues were treated as cations under physiological conditions. Next, a free glycine included in the crystal structure was removed, and the initial coordinates of hydrogen atoms of crystal waters (one water, WAT620, was removed, since the initial coordinate of the hydrogen atoms could not be assigned) were predicted using HMHC. Subsequently, partial optimization by Belly calculations for all components, except heavy atoms, was performed, and distance restraint conditions (7.0 kcal·mol^−1^·Å^−2^) were applied to all heavy atoms of the above structure; next, geometry optimization calculations were performed. The resulting structure was subjected to low‐temperature molecular dynamics annealing (starting temperature 0 K; heat time 30 ps; simulation temperature 300 K; run time 100 ps; final temperature 0 K; cooling time 30 ps; step size 0.001 ps; and temperature step 0.01 K). Finally, all distance restraint conditions were removed, and the structure was further optimized to obtain the final structure. Precision of the final structure was confirmed using a Ramachandran plot program of HMHC.

### Preparation of 3D structures from the ChEMBL database

Planar structures of the ChEMBL database (ChEMBL26; 1 950 760 distinct compounds, including 13 308 drugs) [4] were downloaded from the ChEMBL website in SDF file format. MayaChemTools (2019) [8] was used to remove counterions and inorganic compounds from the database. Then, 3D structures were obtained using balloon version 1.6.9 [9] under an MMFF94 force field. The resulting 3D structure database was treated using babel version 2.4.1 [10]; the compounds’ protonation state was prepared under physiological conditions (pH = 7.4) and filtered by molecular weight (MW ≥ 100 and ≤ 500) to reduce the database to a more drug‐like library. A total of 1 485 144 compounds were used for subsequent virtual screenings.

### Structure‐based virtual screenings

Structure‐based virtual screenings were performed using rdock (2013) [11] and autodock vina version 1.1.2 [12]; both interfaces are available in Docking Study with hyperchem (DSHC) software [5, 13], and the resulting docking modes filtered by the rdock score threshold were more precisely simulated using autodock vina.

Prior to docking simulations, the inhibitor of the A chain of the M^pro^ homodimer was removed from the system. Then, docking simulations for the 3D structure database (1 485 144 compounds) were performed using the relatively reliable, high‐speed docking simulation program rdock under the default condition. I expected that the concentrated potential drugs would consist of ~ 70 distinct drugs, the others being bioactive compounds. On the basis of this, I determined the rdock score threshold to be ≤ −50 kcal·mol^−1^. The cavity condition for rdock docking simulations was prepared using peptidomimetic α‐ketoamide inhibitor of the A chain under a default condition. Docking simulations output the top three docking modes per trial compound in SDF file format. As a result, 4 455 432 docking modes were obtained. These docking modes were filtered by the rdock score threshold of ≤ −50 kcal·mol^−1^ to obtain 27 561 distinct compounds (57 649 docking modes) in SDF file format. The ChEMBL IDs of these distinct compounds were subjected to knime version 4.1.2 [14] to collect compound information from the ChEMBL web server; some information was manually collected from the Kyoto Encyclopedia of Genes and Genomes (KEGG) database [15].

From the 57 649 docking modes obtained by virtual screenings, the 27 561 distinct hit compounds had two docking modes on average. The hit compounds, including the 64 drugs I found, could be more precisely investigated using autodock vina docking simulations with these docking modes as the initial structure. Subsequently, the resulting 57 649 docking modes were separated and converted into individual PDBQT files using DSHC. Then, more precise docking simulations were performed using the autodock vina *In Silico* Screenings interface integrated into DSHC. The M^pro^ homodimer system prepared above in PDB file format was also converted to a PDBQT file using DSHC. A configuration file with cavity information was prepared using DSHC, and other docking conditions were set to default values (the top nine docking modes per trial compound were maximally outputted). Docking simulations with autodock vina produced 513 597 docking modes, which were filtered by the autodock vina score (empirical binding free energy) threshold of −10 kcal·mol^−1^. Since the autodock vina score is an empirical binding free energy, I expected that −9 kcal·mol^−1^ of a score would theoretically show an nM order of binding affinity with M^pro^. When the threshold for screening was set to less than this value, I obtained 659 distinct compounds (1216 docking modes) as hit compounds. To more realistically concentrate the number of hit compounds, I determined the threshold value to be ≤ −10 kcal·mol^−1^. As a result, I obtained 29 distinct compounds (total 41 docking modes). The ChEMBL IDs of these distinct compounds were subjected to KNIME to collect compound information from the ChEMBL web server.

## Results and Discussion

### Structure‐based virtual screenings of the ChEMBL database

In the ChEMBL database, drugs, including approved, clinical, and preclinical drugs, constitute ~ 0.7% of the total number of compounds; the others are mainly bioactive compounds, whose synthesis is, therefore, promising. The advantage for using the ChEMBL database is that it covers all types of drugs, from preclinical to approved stages. I expected that the hit compounds would largely differ from candidates obtained from virtual screenings using focused and targeted libraries [16, 17]. With regard to drug repositioning, the ChEMBL database is more suitable for searching for effective known drugs or bioactive compounds when urgent therapy is necessary and effective drugs are not known. The rdock score threshold of ≤ −50 kcal·mol^−1^ showed relatively high binding affinity with M^pro^.

Table 1 shows the 64 potential drugs that showed high binding affinity with M^pro^, with some drug information collected from the ChEMBL web server using KNIME. I found 11 approved, 14 clinical, and 39 preclinical drugs from the hit compounds (27 561 distinct compounds with 57 649 docking modes); the other 27 497 were bioactive compounds. The 64 drugs were largely classified into antibacterial, antidiabetic, anti‐infective, anti‐inflammatory, antineoplastic, cardiovascular, gastrointestinal, human immunodeficiency virus, and neuropsychiatric drugs. Interestingly, the potential drugs obtained contained sepimostat and curcumin, which are recommended as potential anti‐SARS‐CoV‐2 drugs by researchers [18, 19].

**Table 1:** Potential anti‐SARS‐CoV‐2 drugs obtained from rdock virtual screenings of the ChEMBL database.

CHEMBL ID	Drug synonym	Stage	Action	Target	rdock Score (kcal·mol^−1^)	Vina Score (kcal·mol^−1^)
CHEMBL2105088	LOBENDAZOLE		Anthelmintic		−52.1429	−6.5
CHEMBL2105653	SETILEUTON		Antiasthmatic	5‐Lipoxygenase inhibitor	−60.4636	−8.3
CHEMBL1191	SULFAMETHIZOLE	Approved	Antibacterial	Dihydropteroate synthase inhibitor	−79.7939	−6.6
CHEMBL437	SULFATHIAZOLE	Approved	Antibacterial	Dihydropteroate synthase inhibitor	−72.0537	−6.5
CHEMBL1384	KANAMYCIN	Approved	Antibacterial	30S ribosomal subunit inhibitor	−71.2391	−7.5
CHEMBL1747	TOBRAMYCIN	Approved	Antibacterial	50S ribosomal subunit inhibitor	−56.0916	−6.6
CHEMBL1524273	PHTHALYLSULFATHIAZOLE	Approved	Antibacterial	Cytochrome P450 3A4, dihydropteroate synthase inhibitor	−51.7695	−7.3
CHEMBL2105399	SULFAMOXOLE		Antibacterial	Dihydropteroate synthase inhibitor	−87.8995	−7.2
CHEMBL1355299	SULFAETHIDOLE		Antibacterial	Putative fructose‐1,6‐bisphosphate aldolase	−84.7512	−7.0
CHEMBL2105398	SULFAMETROLE		Antibacterial		−69.6628	−6.6
CHEMBL2105403	PENTISOMICIN		Antibacterial		−59.2134	−7.3
CHEMBL2110604	BETAMICIN		Antibacterial		−54.6510	−7.7
CHEMBL2107073	SANFETRINEM CILEXETIL		Antibacterial		−52.6940	−7.8
CHEMBL94087	GLYBUTHIAZOL		Antidiabetic		−83.8342	−6.8
CHEMBL490070	BENAXIBINE		Antidiabetic	Monoamine oxidase A	−52.5382	−6.9
CHEMBL2107408	GLYBUZOLE		Antidiabetic, Anti‐Hyperglycemic,		−73.5918	−6.6
CHEMBL2104694	ACEFLURANOL		Antiestrogen		−57.3375	−7.4
CHEMBL1950289	TANZISERTIB	Phase2	Antifibrotic	c‐Jun N‐terminal kinase inhibitor	−60.6067	−8.5
CHEMBL2107669	VIPROSTOL		Antihypertensive	Prostaglandin analogue	−52.3341	−6.5
CHEMBL2106914	PHTHALYLSULFAMETHIZOLE		Anti‐infective		−84.7500	−7.9
CHEMBL2106807	MALEYLSULFATHIAZOLE		Anti‐infective		−66.6682	−7.0
CHEMBL157337	RAMIFENAZONE		Anti‐Inflammatory	Adrenergic receptor beta	−79.4409	−6.3
CHEMBL2104561	ELTENAC		Anti‐Inflammatory	COX2	−72.5029	−6.1
CHEMBL114586	SEPIMOSTAT		Anti‐Inflammatory	Serine protease inhibitor	−58.1205	−7.9
CHEMBL2110642	DIBUPYRONE		Anti‐Inflammatory		−57.8675	−6.1
CHEMBL2104226	ETERSALATE		Anti‐Inflammatory		−53.3912	−7.0
CHEMBL2058833	GANAPLACIDE	Phase2	Antimalarial		−70.6688	−7.7
CHEMBL2396661	ALPELISIB	Approved	Antineoplastic	Serine‐protein kinase ATM	−67.1970	−8.3
CHEMBL25336	BISANTRENE	Phase3	Antineoplastic		−54.2373	−8.5
CHEMBL2103842	VARLITINIB	Phase2	Antineoplastic	EGFR‐HER2 inhibitor	−69.1763	−8.1
CHEMBL2180604	TAK‐593	Phase1	Antineoplastic	Vascular endothelial growth factor receptor 3	−65.4614	−8.1
CHEMBL3182444	MK‐5108	Phase1	Antineoplastic	Aurora‐A kinase inhibitor	−52.9359	−6.7
CHEMBL1079	TIZANIDINE	Approved	Cardiovascular	Adrenergic receptor alpha agonist	−78.7516	−6.3
CHEMBL259223	MENATETRENONE	Phase3	Cardiovascular	Vitamin K‐dependent gamma‐carboxylase	−75.9905	−6.3
CHEMBL321582	BUCINDOLOL	Phase2	Cardiovascular	Adrenergic receptor beta antagonist	−50.6285	−7.0
CHEMBL12552	BIMAKALIM		Cardiovascular	Potassium channel opener	−67.8339	−7.1
CHEMBL2106134	DALBRAMINOL		Cardiovascular	Beta blocker	−67.3284	−6.3
CHEMBL358373	INDANIDINE		Cardiovascular	Adrenergic receptor alpha agonist	−66.5682	−6.2
CHEMBL297362	XYLAZINE		Cardiovascular	Adrenergic receptor alpha agonist	−53.0909	−5.7
CHEMBL689	MANNITOL	Approved	Gastrointestinal		−51.6980	−5.3
CHEMBL70209	ZALTIDINE		Gastrointestinal	Histamine receptor H2 antagonist	−57.8372	−6.3
CHEMBL1742413	PIBUTIDINE		Gastrointestinal	Histamine 2 receptor antagonist	−53.1955	−7.7
CHEMBL116438	CURCUMIN	Phase3	HIV	HIV‐1 integrase	−55.7724	−7.3
CHEMBL2360841	RO‐24‐7429	Phase2	HIV	Tyrosyl‐DNA phosphodiesterase 1	−58.6922	−6.7
CHEMBL2105488	THYMOTRINAN		Immunostimulant		−50.6933	−7.1
CHEMBL593262	PARA‐NITROSULFATHIAZOLE		Leishmania Infantum		−80.0130	−7.0
CHEMBL2107425	GLUCUROLACTONE		Liver function improving		−50.5937	−5.8
CHEMBL1108	DROPERIDOL	Approved	Neuropsychiatric	Dopamine D2‐receptor antagonist	−59.2556	−7.5
CHEMBL1522	ESZOPICLONE	Approved	Neuropsychiatric	GABA‐A receptor agonist	−54.5048	−10.0
CHEMBL1618018	HOMATROPINE	Approved	Neuropsychiatric	Muscarinic cholinergic receptor antagonist	−50.4433	−6.7
CHEMBL1394756	ESOXYBUTYNIN		Neuropsychiatric	NF‐Kappa‐B, muscarinic cholinergic receptor antagonist	−51.7716	−5.9
CHEMBL2110912	DIHEXYVERINE		Neuropsychiatric	Muscarinic cholinergic receptor antagonist	−51.2083	−6.8
CHEMBL55214	NERIDRONIC ACID	Phase3	Osteogenesis Imperfecta		−52.9425	−5.6
CHEMBL2106834	METOXEPIN		Psychotropic		−53.3412	−7.4
CHEMBL1231124	AZD‐1480	Phase2		Tyrosine‐protein kinase JAK2 inhibitor	−56.3449	−8.0
CHEMBL10188	TALNETANT	Phase2		Neurokinin 3 receptor antagonist	−52.4637	−7.7
CHEMBL563646	EVATANEPAG	Phase2		Prostanoid EP2 receptor	−50.5628	−8.0
CHEMBL2105528	BISFENAZONE			Carboxylesterase	−66.3130	−7.9
CHEMBL2105110	LAMTIDINE			Histamine 2 receptor antagonist	−65.9473	−6.9
CHEMBL67654	CAREBASTINE			Histamine H1 receptor antagonist	−55.9690	−7.7
CHEMBL155674	ASOBAMAST			TNF receptor 2	−52.7795	−7.1
CHEMBL1603949	BITHIONOLOXIDE			Menin/histone‐lysine *N*‐methyltransferase MLL	−52.4736	−6.9
CHEMBL2105536	SULFACECOLE				−52.0995	−7.0
CHEMBL2104446	VANYLDISULFAMIDE				−50.1930	−8.3

### Additional docking simulations for hit compounds

Table 2 shows the 29 hit compounds obtained using autodock vina virtual screenings with ≤ −10 kcal·mol^−1^ of binding free energy for M^pro^. For the 64 drugs, autodock vina scores of the most stable docking modes are also shown in Table 1.

**Table 2:** Hit compounds obtained by combining autodock vina and rdock virtual screenings of the ChEMBL database.

CHEMBL ID	Structure	Target	Vina score (kcal·mol^−1^)
CHEMBL1559003	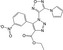	Survival motor neuron protein	−10.6
CHEMBL2237553	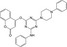	*Aspergillus niger*	−10.5
CHEMBL1511674	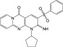	Histone‐lysine *N*‐methyltransferase MLL	−10.5
CHEMBL3260476	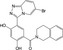	Heat shock protein HSP 90‐alpha	−10.4
CHEMBL1170272	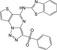	Serotonin 6(5‐HT6) receptor	−10.4
CHEMBL1335000	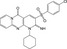		−10.4
CHEMBL2235580	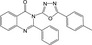	*Mus musculus*	−10.3
CHEMBL3264032	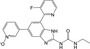	*Staphylococcus aureus*	−10.3
CHEMBL1447105	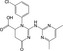	4′‐phosphopantetheinyl transferase FFP	−10.2
CHEMBL589899	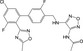	Bradykinin B1 receptor	−10.2
CHEMBL1539803	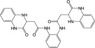	Lysine‐specific demethylase 4D‐like	−10.2
CHEMBL2216842	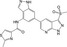	PI3‐kinase p110‐delta subunit	−10.2
CHEMBL427787	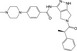	Serine threonine‐protein kinase aurora‐A	−10.2
CHEMBL1339675	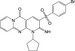		−10.2
CHEMBL3126648	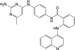	DNA(Cytosine‐5)‐methyltransferase 1	−10.1
CHEMBL1302388	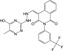	Prelamin‐A/C	−10.1
CHEMBL3234783	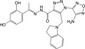	*Staphylococcus aureus*	−10.1
CHEMBL1807774	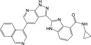	Tyrosine‐protein kinase receptor RET	−10.1
CHEMBL2087984	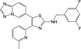		−10.1
CHEMBL2387487	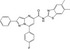	ACHN	−10.0
CHEMBL2113271	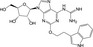	Adenosine A1 receptor	−10.0
CHEMBL476947	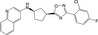	Cannabinoid CB2 receptor	−10.0
CHEMBL399042	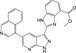	Cyclin‐dependent kinase 1	−10.0
CHEMBL2000247	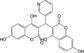	Integrase	−10.0
CHEMBL3236740	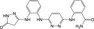	*Mus musculus*	−10.0
CHEMBL1447944	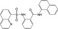	Nonstructural protein 1	−10.0
CHEMBL1760165	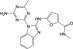	Serine threonine‐protein kinase mTOR	−10.0
CHEMBL2087965	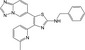		−10.0
CHEMBL1522	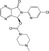	GABA‐A receptor agonist	−10.0

Almost all the 29 hit compounds were bioactive compounds registered to the ChEMBL database, except for eszopiclone (CHEMBL1522; approved drug for neuropsychiatric disease). I believe that these hit compounds were not developed for common targets, although the structural feature could be categorized into some mother skeletons, such as diazole, azine, and sulfone derivatives (Table 2).

**Fig. 2:**
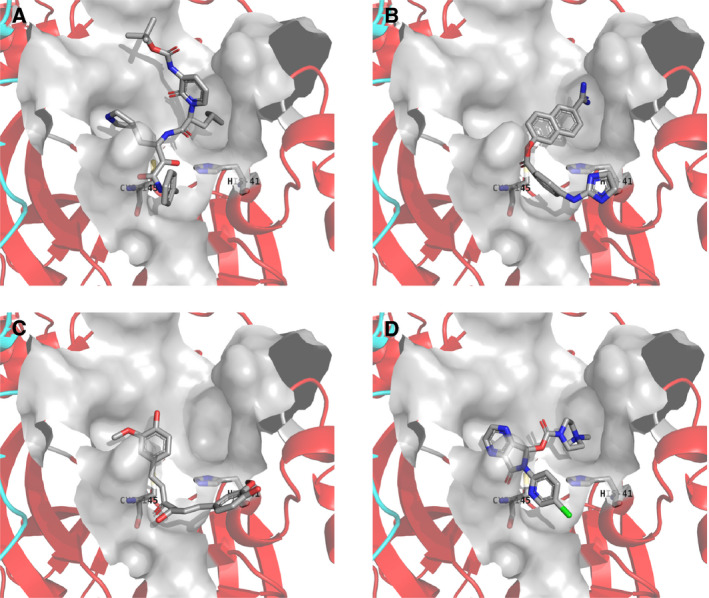
The most stable docking mode obtained from autodock vina docking simulations. (A) Binding mode of peptidomimetic α‐ketoamide inhibitor in the crystal structure. (B) Sepimostat, (C) curcumin, and (D) eszopiclone. Chains A and B of the M^pro^ homodimer are shown as red and cyan ribbons, respectively. The compound, Cys145, and His41 are shown as tubes. Van der Waals surface of the active site is shown in gray color. Water molecules and hydrogen atoms are neglected.

Figure 2 shows the most stable docking modes of sepimostat (Fig. 2B; autodock vina score −7.9 kcal·mol^−1^), curcumin (Fig. 2C; autodock vina score −7.3 kcal·mol^−1^), and eszopiclone (Fig. 2D; autodock vina score −10.0 kcal·mol^−1^) obtained from autodock vina docking simulations, in addition to the binding mode of peptidomimetic α‐ketoamide inhibitor in the crystal structure (Fig. 2A). Researchers consider sepimostat, curcumin, and the α‐ketoamide inhibitor to be potential anti‐SARS‐CoV‐2 drugs. In this study, eszopiclone was also the only approved drug with the highest score on autodock vina docking simulations (Tables 1 and 2). The carbonyl moiety of these compounds was close to the catalytic site of the Cys145 and His41 catalytic dyad, and docking modes were similar to each other. These results suggest that these compounds may function through the same underlying mechanism.

## Conclusions

This study was performed to rapidly identify potential anti‐SARS‐CoV‐2 drug candidates from a known drug library on the basis of drug repositioning. The drug candidates presented in this study could be further examined for their anti‐SARS‐CoV‐2 activities, together with those of earlier studies using more limited drug libraries [20, 21, 22]. Bioactive compounds with high binding affinity for SARS‐CoV‐2 M^pro^ could be used as a basis for improving pharmacokinetic properties and for developing specific anti‐SARS‐CoV‐2 drugs. Combinations of structure‐based docking simulations are valuable for high‐throughput virtual screenings to identify urgently needed therapies for viral infections. Determination of the effect of potential anti‐SARS‐CoV‐2 drugs obtained in this study is in progress, and the results will be published in the near future.

## Conflict of interest

The author declares no conflict of interest.

## Author contributions

MT designed the experiments, prepared the infrastructure of the experiments, developed the software, performed the experiments, analyzed the data, and wrote the paper.
